# Attitudes, Behaviors, and Barriers among Adolescents Living with Obesity, Caregivers, and Healthcare Professionals in Spain: ACTION Teens Survey Study

**DOI:** 10.3390/nu15133005

**Published:** 2023-06-30

**Authors:** Juan Pedro López Siguero, Marta Ramon-Krauel, Gilberto Pérez López, Maria Victoria Buiza Fernández, Carla Assaf Balut, Fernando Fernández-Aranda

**Affiliations:** 1 Pediatric Endocrinology Unit, Regional University Hospital, 29011 Málaga, Spain; 2 Department of Endocrinology, Institut de Recerca Sant Joan de Déu, Hospital Sant Joan de Déu, 08950 Barcelona, Spain; marta.ramon@sjd.es; 3 Spanish Biomedical Research Centre in Diabetes and Associated Metabolic Disorders (CIBERDEM), Health Institute Carlos III, 28029 Madrid, Spain; 4 Department of Endocrinology and Nutrition, Hospital Gregorio Marañon, 28007 Madrid, Spain; beto_med@hotmail.com; 5 Bariatric Association Hispalis, ABHispalis, 39009 Santander, Spain; abhispalis.secretaria@gmail.com; 6 Medical Affairs, Novo Nordisk Spain, 28033 Madrid, Spain; nczb@novonordisk.com; 7 Clinical Sciences Department, School of Medicine and Health Sciences, University of Barcelona, 08907 Barcelona, Spain; ffernandez@bellvitgehospital.cat; 8 Clinical Psychology Unit, University Hospital of Bellvitge-IDIBELL, 08907 Barcelona, Spain; 9 Spanish Biomedical Research Center in Obesity and Nutrition (CIBEROBN), Health Institute Carlos III, 28029 Madrid, Spain

**Keywords:** adolescents living with obesity, barriers, clinical care, obesity management, physician attitudes

## Abstract

Although the prevalence of pediatric obesity is rising, understanding of the perceptions, attitudes, behaviors, and barriers to effective obesity care among Spanish adolescents living with obesity (ALwO), their caregivers, and healthcare professionals (HCPs) is lacking. In 2021, the cross-sectional ACTION Teens survey study was conducted in 10 countries; results from the Spanish cohort are presented herein. The survey was completed by 648 ALwO, 644 caregivers, and 251 HCPs in Spain. A total of 25% of ALwO and 43% of caregivers thought that their/their child’s weight was normal, and more caregivers than ALwO perceived the ALwO’s health to be at least good (95% vs. 59%, respectively). Only 53% of ALwO and 9% of caregivers reported receiving an obesity diagnosis, despite HCPs reporting they provide diagnoses to 87% of ALwO/caregivers. Although 65% of HCPs felt that ALwO may not be comfortable discussing weight, only 26% of ALwO who had discussed weight with an HCP (*n* = 488) reported not feeling comfortable. Inability to control hunger was a key barrier to ALwO losing weight identified by ALwO/caregivers, but not HCPs. Improved communication between the three groups, a better understanding of barriers to weight loss, and improved health education on obesity are needed in order to enhance obesity care in Spain.

## 1. Introduction

Pediatric obesity is a major health concern globally [1]. The prevalence of childhood and adolescent obesity has grown steadily in Spain in recent years. A meta-analysis of studies evaluating the prevalence of overweight/obesity among Spanish children demonstrated an increase among those aged 7–13 years, from 32.3% in 1999–2010 to 35.3% during 2011–2021 [2]. Preliminary results from Physical Activity, Sedentarism, and Obesity of Spanish youth (PASOS) 2022, a study on 2892 Spanish children aged 8–16 years, revealed the prevalence of overweight/obesity to be 33.4%, obesity 11.8%, and, notably, abdominal obesity 20.4%; this represented a 1.3% increase in obesity from 2000 to 2022 [3].

Childhood and adolescent obesity are associated with multi-organ system morbidity, which includes metabolic and musculoskeletal disturbances, cardiovascular comorbidities, and pulmonary complications, in addition to greater risk of premature death [4]. Accordingly, childhood/adolescent obesity is associated with a greater prevalence of cardiometabolic risk factors and with changes in the heart, including enlarged heart chambers, increased left ventricular mass, and decreased left ventricle ejection fraction [5,6]. In addition to physical comorbidities, children with excess weight are more prone to psychosocial distress than children of a healthy weight; this may be further impacted by bullying/teasing and the stigma of childhood obesity [7]. Childhood obesity increases the risk of obesity in adolescence, which then leads to a greater risk of obesity in adulthood [5]. Approximately 80% of those with obesity during adolescence will have obesity when they become adults, which decreases to around 70% when only considering adults aged >30 years [8]. Childhood and adolescent obesity are associated with a greater risk of obesity-related morbidities in adulthood, including cardiovascular disease, diabetes, and some cancers, as well as a greater risk of cardiovascular death [5,9,10]. 

Intervention at younger ages is associated with greater long-term decreases in body mass index (BMI) and larger reductions in the risk of future diseases, including cardiovascular conditions, type 2 diabetes, and certain cancers [4,11]. Consequently, the timely diagnosis/treatment of obesity in children and adolescents should be prioritized. Although there is now a better understanding of the complexities of obesity, treatment outcomes for childhood and adolescent obesity remain suboptimal [4,12]. Despite this, studies analyzing underlying obstacles to effective obesity management in adolescents living with obesity (ALwO) are scarce, and there are key evidence gaps in terms of the lived experiences, unmet needs, and challenges of ALwO, their caregivers, and their healthcare professionals (HCPs). A survey study in 11 countries that evaluated behaviors, attitudes, and perceptions of adults with obesity and HCPs reported a propensity for HCPs to delay the initiation of weight management conversations until complications develop [13]. As such, there remains a need to start conversations about weight at an early stage of patient management, before the development of obesity-related complications. 

The Awareness, Care, and Treatment In Obesity maNagement (ACTION) Teens study was a global, cross-sectional survey study in 10 countries that aimed to identify behaviors, attitudes, perceptions, and barriers to effective obesity care among ALwO, caregivers of ALwO, and HCPs who treat ALwO [14]. ACTION Teens reported misalignment between the three groups, including a lack of understanding of the effect of obesity on ALwO by caregivers and misperception by HCPs of the motivators and barriers for their patients to lose weight [14]. Herein, we present ACTION Teens data from Spain and discuss the similarities and differences to the global ACTION Teens study. Identifying country-specific behaviors, attitudes, perceptions, and obstacles to obesity care will provide input for strategies with which to improve management of ALwO in Spain. 

## 2. Materials and Methods

### 2.1. Study Design and Participants

ACTION Teens (ClinicalTrials.gov identifier: NCT05013359) was a cross-sectional, online survey study that collated data from three groups of respondents (ALwO, caregivers, and HCPs) in 10 countries/regions (Australia, Colombia, Italy, Mexico, Saudi Arabia, South Korea, Spain, Taiwan, Turkey, and the United Kingdom). The full methods for ACTION Teens have been published previously [14]. Participants from Spain were surveyed between August and December 2021 (ALwO and caregivers) or between August and September 2021 (HCPs). 

For the Spanish cohort, the recruited ALwO were 12 to <18 years old and living in Spain. Obesity was defined as a BMI (calculated from self-reported height, weight, sex, and age) ≥95th percentile for sex and age based on World Health Organization charts (2007) [15]. The recruited caregivers were ≥25 years old, resided (≥50% of the time) with an ALwO in Spain, and participated in healthcare decisions for the ALwO. The recruited HCPs were required to be practicing in Spain and to have been in clinical practice for 2 or more years. They were also required to spend ≥50% of their time directly caring for patients and to see 10 or more ALwO per month. 

### 2.2. Survey Development

Three distinct but overlapping surveys (one each for ALwO, caregivers, and HCPs) were developed. A steering committee comprising content experts and HCPs co-developed and approved all survey materials. Translation and back-translation from the English version were conducted and accuracy was fulfilled. The questionnaires have been published previously in the Online Supporting Information section of the global ACTION Teens study manuscript [14]. 

### 2.3. Procedures

KJT Group Inc. (Rochester, NY, USA) oversaw data acquisition and reporting. An online survey utilizing Decipher Survey software (FocusVision Worldwide Inc., Stamford, CT, USA) was used for data collection. Online panels/databases were used to recruit caregivers and ALwO by targeting and screening adults from a stratified general population sample to identify caregivers of an ALwO. “Matched pairs” of ALwO and caregivers were maximized by asking verified caregivers for permission to enroll their ALwO. Following the maximization of the matched pairs, additional ALwO and caregivers were recruited to obtain the target sample size. HCPs were recruited from online physician panels/databases. Surveys were provided in Spanish and completed online by respondents or via in-person interviews (caregivers and ALwO only).

### 2.4. Outcomes

As described previously [14], the primary outcome measures included the following: attitudes about people with obesity and obesity, and beliefs related to the impact of obesity; attempts to lose weight in the last year, barriers/motivations to losing weight, and definition of successful weight loss/management; frequency/history of weight conversations including the initiator, and responsibility for starting conversations about weight between HCPs and ALwO/caregivers; interactions between HCPs, ALwO, and caregivers, frequency of diagnosing obesity, reasons for not discussing obesity, and follow-up appointments; and information sources for learning about healthy lifestyles, weight loss/management, and obesity. Five-point Likert scales, yes/no responses, numeric responses, and single/multiple item selection were used to assess outcome measures, as appropriate. 

### 2.5. Sample Size

The target number of completed surveys for Spain was 650 for ALwO, 650 for caregivers, and 250 for HCPs. These sample sizes were chosen to ensure statistical power while maintaining recruitment feasibility.

### 2.6. Statistical Analysis

As described previously [14], the full analysis set comprised all those who answered the survey in its entirety. KJT Group Inc. analyzed de-identified data using Stata (version IC 14.2; StataCorp LLC, College Station, TX, USA), SPSS (version 23.0; IBM, Armonk, NY, USA) and Excel (Microsoft). Univariate descriptive statistics (proportions, medians, and means) were used to summarize data. For continuous variables, where appropriate, outliers were truncated to the median. For generalizability and mitigation of selection bias, caregiver data were weighted to demographic targets representative for Spain for region, sex, age, household income, and education. Demographic targets were aligned with Spanish census data and were based on data from the United States Census Bureau–International Data Base, World Education News and Reviews (WENR), and Instituto Nacional de Estadística (INE). The target adult general population sample was stratified to match these general population targets to ensure that the qualifying sample was largely representative of the adult population in Spain. The final incoming sample for the adult general population (i.e., the final caregiver sample), which included those failing to qualify for the survey, was then weighted to match the demographic targets. To obtain the nearest possible target and sample balance, weights were calculated using a raking technique; individual respondent weights were capped at 0.5 and 5.00 to avoid extreme design effects.

## 3. Results

### 3.1. Participant Characteristics

A total of 648 ALwO, 644 caregivers, and 251 HCPs were surveyed in Spain (Figure S1). The HCP response rate was 16.8%. Due to the recruitment methods, calculation of ALwO and caregiver response rates was not possible [14]. 

Table 1 shows the demographics and characteristics of the participants. Just over one-third (36%) of the ALwO were female, while almost half of the caregivers (45%) and HCPs (46%) were female. Approximately half (53%) of the ALwO and just over one-third (36%) of the caregivers indicated that the mother or father of the ALwO had overweight. Approximately 60% of the caregivers who answered the survey had overweight/obesity, and HCPs reported that 68% of their ALwO patients had family members with obesity in the household. HCPs’ primary medical specialty and professional experience are reported in Table S1.

### 3.2. Information Sources

The information sources most commonly used by ALwO to learn about weight loss, healthy lifestyles, and weight management were YouTube, family and friends, information from a doctor, and social media, while for caregivers, they were information from a doctor, family and friends, YouTube, and dietitian/nutritionist (non-doctor) (Figure S2). For HCPs, the most commonly used sources of information were medical education/continuing medical education programs, journal articles, and conferences (Figure S2).

### 3.3. Perceptions of Obesity

**Figure 1:**
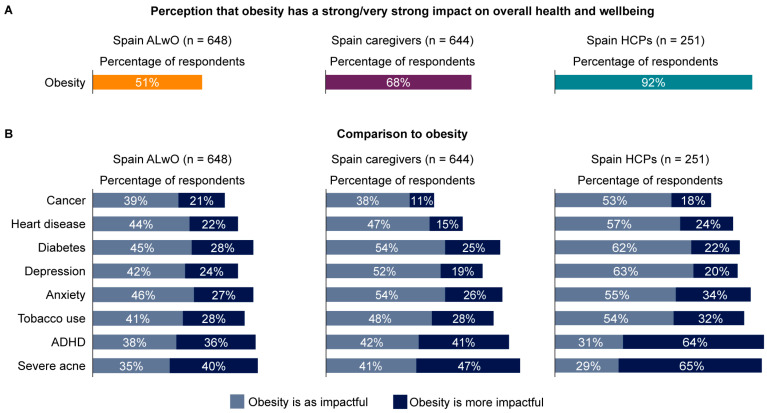
Perceived impact of obesity on overall health and wellbeing among ALwO, caregivers, and HCPs: (**A**) impact of obesity; (**B**) impact of obesity compared with other health conditions. Participants were asked how much of an impact they thought various health conditions had on a person’s overall health and wellbeing using the following scale: 1 = no impact, 2 = slight impact, 3 = moderate impact, 4 = strong impact, and 5 = very strong impact (ALwO/caregiver Q510; HCP Q305). Panel A represents the proportion of respondents who indicated that obesity has a strong/very strong impact. Data were recoded to compare each participant’s response regarding the impact of obesity with their response regarding the impact of other health conditions, and are shown in panel B; if the response was higher for obesity than for another health condition, it was coded as “Obesity is more impactful”; if equal, it was coded as “Obesity is as impactful”. ADHD, attention deficit hyperactivity disorder; ALwO, adolescents living with obesity; HCP, healthcare professional. Figure adapted from [14].

HCPs were more likely than caregivers or ALwO to believe that obesity has a strong/very strong impact on overall health and wellbeing (Figure 1). When evaluating the respondents’ perceptions of the impact of obesity compared with other comorbidities, most respondents in the ALwO, caregiver, and HCP groups considered obesity to be as impactful or more impactful than several other health conditions, including diabetes, heart disease, and depression (Figure 1). 

### 3.4. Impact of Obesity

**Figure 2:**
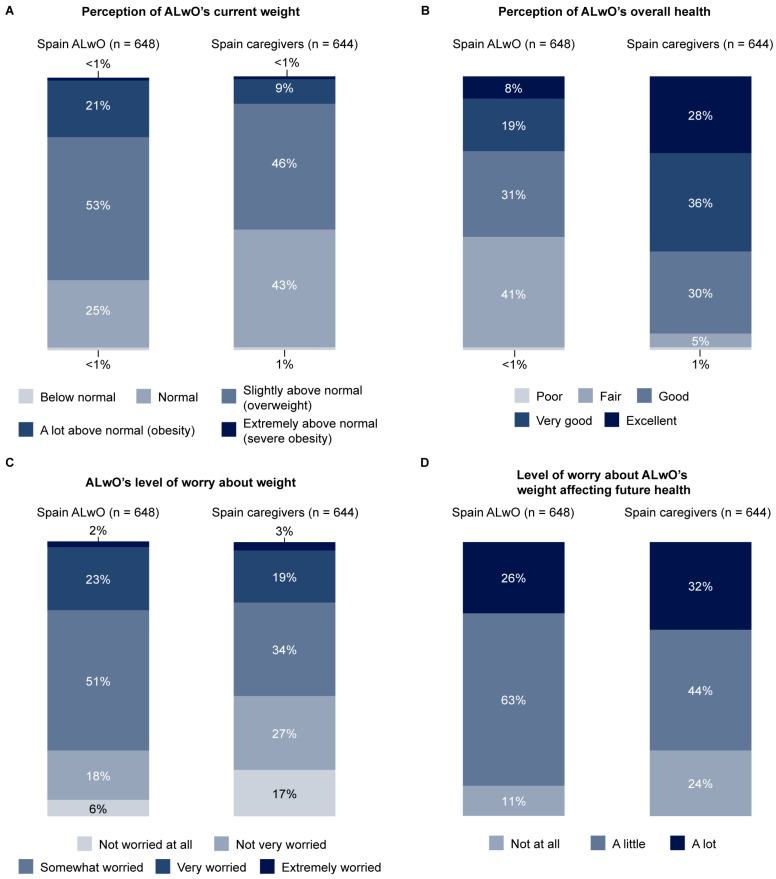
Perceptions of the impact of obesity on ALwO: (**A**) ALwO and caregiver perception of ALwO’s current weight; (**B**) ALwO and caregiver perception of ALwO’s overall health; (**C**) ALwO and caregiver perception of ALwO’s level of worry about weight; (**D**) ALwO and caregiver level of worry about ALwO’s weight affecting future health. Percentages represent the proportions of participants who selected each prespecified response option among all recruited ALwO (left bars) and caregivers (right bars). Percentages may not sum to 100% due to rounding. ALwO data are based on responses to ALwO Q106, Q101, Q108, and Q512, and caregiver data are based on responses to caregivers Q106, Q101, Q112, and Q515. ALwO, adolescents living with obesity. Figure adapted from [14].

Most ALwO (53%) considered their weight to be slightly above normal (overweight), while 25% of ALwO thought their weight was normal (Figure 2). However, caregivers were more likely to think that the weight of their ALwO was normal (43%) (Figure 2). Considering their overall health, 59% of ALwO believed it to be at least good. In contrast, 95% of caregivers believed that their ALwO’s health was at least good (Figure 2). Only 1% or fewer ALwO and caregivers considered their/their child’s health to be poor (Figure 2).

Regarding how much the ALwO worried about their weight, 25% were very or extremely worried and 76% were at least somewhat worried (Figure 2). A similar percentage of caregivers reported that their ALwO was very or extremely worried about their weight (22%), but fewer reported that their ALwO was at least somewhat worried (56%) (Figure 2). More ALwO than caregivers worried a lot or a little about the ALwO’s weight affecting their future health (Figure 2).

### 3.5. Weight Loss

**Figure 3:**
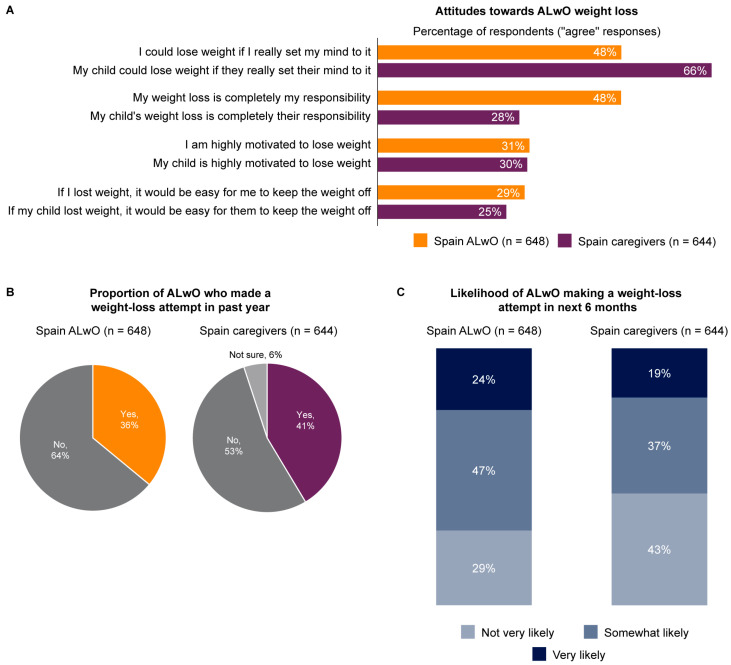
Weight loss attitudes and ALwO weight loss attempts: (**A**) ALwO and caregiver attitudes towards ALwO weight loss; (**B**) proportion of ALwO who attempted to lose weight in the previous year, according to the ALwO and caregivers; (**C**) likelihood of a weight loss attempt by ALwO in the coming 6 months, according to the ALwO and caregivers. Percentages are proportions of participants among all recruited ALwO or caregivers. ALwO data are based on responses to ALwO Q113, Q108a, and Q109, and caregiver data are based on responses to caregivers Q113, Q110a, and Q111. For each question, response options were prespecified, and only one option could be selected. Panel A presents the proportion of participants who indicated that they “strongly agree” or “somewhat agree” with each statement. For panel C, the “not very likely” category includes the response options “not likely at all” and “not very likely”, and the “very likely” category includes the response options “very likely” and “extremely likely”. Percentages may not sum to 100% due to rounding. ALwO, adolescents living with obesity. Figure adapted from [14].

Overall, 39% of caregivers believed that their child will naturally slim down as they grow older, compared with 29% of HCPs (Figure S3). A greater proportion of caregivers than ALwO agreed that the ALwO could lose weight if he/she set their mind to it (Figure 3). More ALwO (48%) than caregivers (28%) felt that weight loss was completely the responsibility of the ALwO (Figure 3), while 21% of HCPs thought that losing weight was completely their patient’s responsibility (Figure S3). Similar proportions of ALwO (36%) and caregivers (41%) indicated that they or their child had attempted to lose weight in the past year, but more ALwO (71%) reported they were somewhat or very likely to try to lose weight within 6 months than caregivers (57%) reported for their ALwO (Figure 3). 

**Figure 4:**
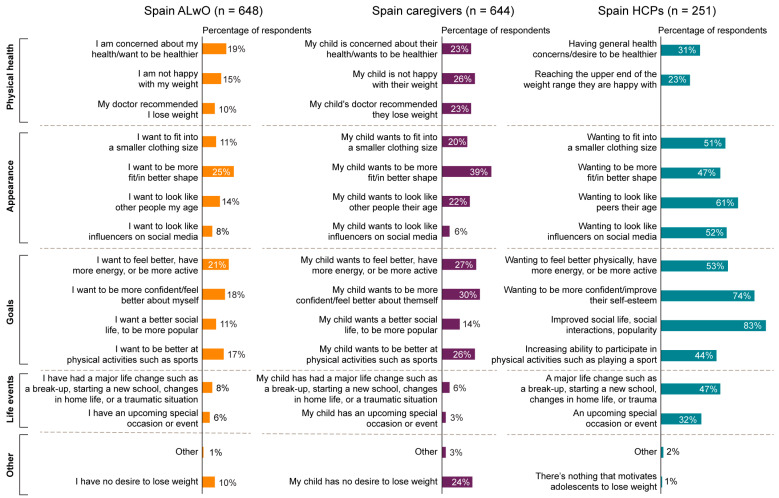
Motivators for ALwO weight loss, as defined by ALwO, caregivers, and HCPs. Response options selected by ALwO and caregivers when asked why they/their child has wanted to lose weight (ALwO/caregiver Q208) are presented in the first two columns. The prespecified response options selected by HCPs when asked what most motivates adolescents to lose weight (HCP Q205) are presented in the third column. ALwO, adolescents living with obesity; HCPs, healthcare professionals. Figure adapted from [14].

For ALwO and caregivers, the most frequently reported reason why ALwO wanted to lose weight was to be more fit/in better shape, while for HCPs, it was a desire to improve popularity and social life (Figure 4). 

The barriers to ALwO weight loss reported by ALwO and caregivers were similar (Figure S4); the most frequently reported barriers were an inability to control hunger, liking to eat unhealthy food, and lack of motivation. HCPs most commonly agreed that unhealthy eating habits, preference for unhealthy food, and insufficient exercise were barriers to ALwO losing weight, while inability to control hunger ranked 8th (Figure S4). The definitions of success for ALwO weight loss according to ALwO, caregivers, and HCPs are reported in Figure S5. 

### 3.6. Conversations about Weight

Over half (54%) of ALwO felt they could talk honestly about their weight with their mother or father, but only 23% felt this way about conversations with their HCP. The most common ALwO- and caregiver-reported barriers to conversations about weight with an HCP were not seeing their/their child’s weight as a significant medical issue and the ALwO already knowing how to manage their weight (Figure S6). The most frequently reported barrier for ALwO to initiating conversations about their weight, as perceived by HCPs, was the ALwO not feeling comfortable bringing up the topic (65%) (Figure S6).

Approximately half (51%) of caregivers had talked to their child’s doctor about their child’s weight in the course of the last year. Both ALwO and caregivers (who had talked with an HCP about their/their child’s weight in the past year) most frequently reported that the parent/caregiver usually started the conversation (Figure S7); contrary to this, HCPs reported that they initiated weight discussions the majority of the time, but only 29% of HCPs felt they are responsible for starting the conversation (Figure S7).

A greater proportion of ALwO (53%) than caregivers (9%) had been informed about their/their child’s obesity diagnosis by their doctor. HCPs reported that they inform, on average, 87% of ALwO or their caregivers about the adolescent’s obesity diagnosis. Furthermore, 50% of the HCPs reported they always recorded an obesity diagnosis in the medical record, and 37% recorded it most of the time. 

**Figure 5:**
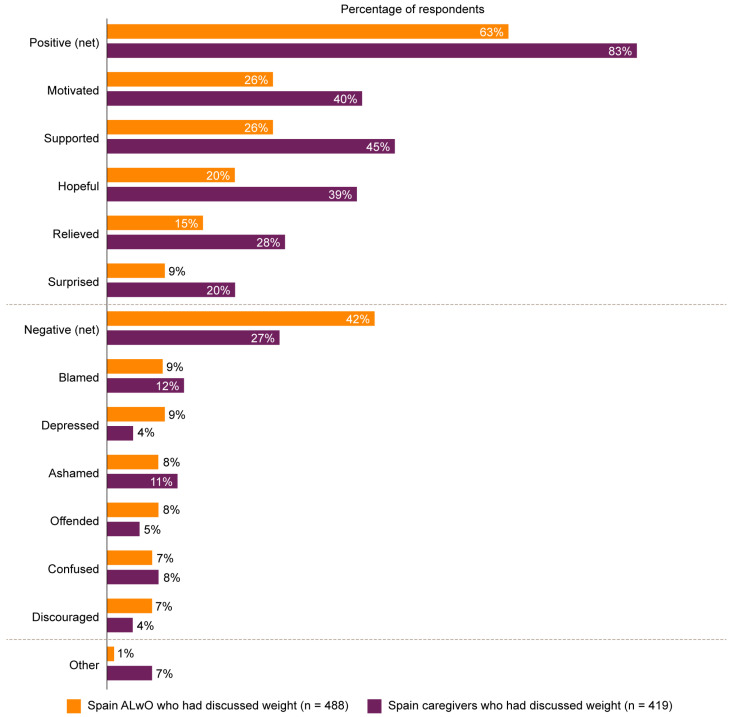
The feelings of ALwO and caregivers following their latest weight discussion with an HCP. Percentages represent the proportions of respondents who selected each of the prespecified response options for ALwO/caregiver Q410, among the subset of ALwO who had discussed weight with an HCP in the past year or the subset of caregivers who had discussed their child’s weight with an HCP in the past year (per ALwO/caregiver Q201). The responses of caregivers represent their own feelings rather than their perception of their child’s feelings. The net positive category is the proportion of participants who selected at least one positive answer (i.e., motivated, supported, hopeful, relieved, and/or surprised); the net negative category is the proportion of participants who selected at least one negative answer (i.e., blamed, depressed, ashamed, offended, confused, and/or discouraged). ALwO, adolescents living with obesity; HCP, healthcare professional. Figure adapted from [14].

Among ALwO (*n* = 488) and caregivers (*n* = 419) who had talked with an HCP about their/their child’s weight in the past year, 40% of ALwO and 71% of caregivers reported feeling comfortable doing so, while 26% and 9%, respectively, were not comfortable participating in these discussions. Most ALwO (63%) and caregivers (83%) reported at least one positive feeling following the latest discussion with an HCP, with the most common feelings reported to be “motivated”, “supported”, and “hopeful” (Figure 5). Among ALwO (*n* = 160) and caregivers (*n* = 225) who had not talked with an HCP about the ALwO’s weight, 44% of ALwO and 55% of caregivers would feel comfortable discussing it.

### 3.7. Weight Management

Most HCPs (87%) believed obesity to be a chronic disease (Figure S8). As reported by ALwO and caregivers, the methods most commonly used by ALwO for managing weight in the past year were becoming more physically active and improving eating habits (Figure S9). The most effective methods for managing weight according to HCPs were becoming more physically active, improving eating habits, and reducing screen time (Figure S9). 

## 4. Discussion

This analysis of the Spanish cohort from the ACTION Teens survey study provides key insights regarding the attitudes, perceptions, and behaviors of ALwO, caregivers, and HCPs, as well as possible obstacles to effective obesity care and Spanish culture-specific factors. There was misalignment between ALwO, caregivers, and HCPs, notably around the perception and impact of obesity and regarding conversations about weight. The findings of this study may change the strategy for interacting with caregivers and adolescents with obesity. The study also highlights the importance of training HCPs not only in biological problems related to obesity, but also in the perceptions, attitudes, and motivations of the people involved.

One-quarter of ALwO and almost half of caregivers considered their/their child’s weight to be normal. The lack of recognition of the ALwO’s obesity status may reflect the relatively small proportion of ALwO (53%) and caregivers (9%) who had been informed of their/their child’s obesity diagnosis by their HCP. In contrast, HCPs stated that they informed the majority (87%) of ALwO/caregivers about the ALwO’s diagnosis of obesity, with almost all reporting that they record obesity diagnoses in medical records most or all of the time. This aligns with the global ACTION Teens study, in which a greater proportion of HCPs reported informing ALwO/caregivers of the ALwO’s obesity diagnosis compared with reports from ALwO/caregivers themselves [14]. This indicates a miscommunication, both in Spain and globally, between HCPs, ALwO, and caregivers with regard to obesity diagnoses, highlighting the importance of education and communicating weight status to ALwO and caregivers. 

Compared with the global cohort, more Spanish caregivers tended to misperceive their child’s health/weight, with a greater proportion perceiving the overall health of their ALwO to be at least good (95% vs. 80%, respectively) and believing the weight of their ALwO to be normal (43% vs. 32%) [14]. This result aligns with a cross-sectional analysis of Spanish school children that reported parental underestimation of the weight category of children with obesity in 94% of cases [16]. The tendency for parents/caregivers to underestimate the overweight status of their children has been reported in other studies worldwide that included Spanish participants [17,18]. The greater propensity among Spanish caregivers, compared with those in the ACTION Teens global cohort, to believe their child’s weight was normal may be related to greater indulgence towards pediatric obesity and failure to recognize the detrimental impact of obesity in Spain. The high proportion of caregivers with overweight or obesity may also have impacted how they perceive the weight of their child. Visual normalization of obesity has been shown to contribute to the perceptions of caregivers, whereby increased exposure to larger body sizes leads caregivers to shift their perception of normal weight in children, leading to an underestimation of the weight of their child [19,20]. Previous data indicate that the lack of perception of obesity by the family is a determining factor in the development or worsening of childhood obesity [21]. Hence, family involvement is considered fundamental to the prevention of childhood obesity [22].

Considering attitudes towards weight loss, many caregivers (39%) thought that their child would slim down with age. This could indicate a misunderstanding of obesity in adolescence and may be an important barrier to seeking support from their HCP for management of their child’s weight. Delayed initiation of medical management may be compounded by views among ALwO and caregivers regarding personal responsibility for weight loss. Approximately one-half of ALwO and one-quarter of caregivers felt that losing weight was solely the ALwO’s responsibility. Feelings of personal responsibility for weight loss among individuals with obesity are not uncommon, arising from a narrative that positions obesity as a choice and/or lack of willpower [23]. Furthermore, when analyzing barriers to weight loss, there was discordance between HCPs and ALwO/caregivers on the importance of the inability to control hunger, with ALwO/caregivers, but not HCPs, reporting this as one of their top three barriers. This may indicate a misunderstanding around the biology of obesity, even among HCPs, and calls for improved education on this topic that can be further communicated to patients. 

There were no remarkable differences between Spanish and global caregivers in terms of key motivators or barriers to weight loss. Key motivators in all respondent groups in Spain and globally included those related to appearance (e.g., being fit and looking like their peers), improved social status (i.e., their social life, interactions, and popularity), and improved self-confidence [14]. The barriers to weight loss most frequently reported by ALwO and caregivers, both in Spain and globally, were the inability to control hunger, lack of motivation, and a preference for unhealthy foods, while HCPs most often reported poor diet and insufficient physical activity [14]. 

Although 88% of Spanish ALwO reported barriers to weight loss in the present study, almost three-quarters of ALwO and over one-half of caregivers indicated that they/their child would be somewhat or very likely to try to lose weight within 6 months. Similar proportions of ALwO (75%) and caregivers (63%) were reported in the global study [14]. As such, HCPs should feel encouraged to initiate conversations about weight with ALwO and caregivers. 

Guidance from the American Academy of Pediatrics (AAP) highlights the importance of communicating weight status to a child with obesity and their family in order to guide the subsequent evaluation and treatment [24]. However, this first step can be challenging for ALwO, caregivers, and HCPs alike, and can present an initial barrier to the advancement of obesity management. Only a small proportion of HCPs believed that they are responsible for initiating conversations about weight. Furthermore, approximately two-thirds of HCPs reported discomfort among ALwO in bringing up the topic of weight. In contrast, only 26% of ALwO who had discussed weight reported a lack of comfort with the conversation. Moreover, most ALwO and caregivers (63% and 83%, respectively) felt positive about previous weight-related discussions with an HCP. Across those who had not previously had a discussion about weight with an HCP, around half of ALwO and caregivers would feel comfortable with the conversation. As such, HCPs should feel confident in raising the topic of weight in order to facilitate effective treatment strategies. Interestingly, while 51% of Spanish caregivers reported having spoken with their child’s doctor about their weight within the last year (compared with 36% of caregivers in the global analysis), less than 10% reported being informed of a diagnosis of obesity (vs. 29% in the global group) [14]. This indicates a miscommunication between HCPs and caregivers that is particularly prominent in Spain compared with other world regions. This may reflect a lack of understanding among Spanish caregivers or an unwillingness of HCPs to engage in challenging discussions.

The recommendations for the prevention and treatment of childhood obesity in Spain are mainly focused on changes in diet and increased exercise, paying little to no attention to the attitudes, behaviors, and motivations of the family and the adolescent, and the importance of these in ensuring successful management of the child’s obesity [25,26,27]. It will be important to integrate the results of this study at the primary care level to facilitate early detection of childhood obesity and provide information about obesity to the family. In Spain, children are checked by the primary pediatrician on numerous occasions during the first years of their lives. As contact with the family is frequent during this time, recommendations related to obesity may have a substantial impact.

The strengths and limitations of this analysis of the Spanish cohort are comparable to those of the overall ACTION Teens study [14]. This was the first evaluation and comparison of the lived experiences of three parties (ALwO, caregivers, and HCPs) involved in obesity management in Spain. This analysis provides valuable information that may be used to align and achieve the objectives of all participating groups. The main limitations of the study include the risk of selection bias and the potential for measurement error due to use of online surveys, the self-reported weight and height data that may have led to underestimation or overestimation of ALwOs’ BMI, the cross-sectional study design, and lack of information on body composition and pubertal stage. Furthermore, only 36% of ALwO in the Spanish cohort were female, and so results may be biased towards male ALwO attitudes, perspectives, and behaviors; subgroup analyses based on sex are outside the scope of this manuscript. The majority of ALwO and caregivers (more than 90%) were not matched. In addition, HCP respondents did not directly care for the surveyed ALwO; therefore, it was not possible to carry out paired studies.

Overall, our findings should be generalizable to the wider population of ALwO, caregivers of ALwO, and HCPs treating ALwO in Spain. Although online surveys typically have poor response rates, broad inclusion and exclusion criteria were used to ensure that respondents were representative of ALwO and HCPs treating ALwO in Spain. Furthermore, stratified sampling and demographic weighting of caregiver data were used to ensure that the sample was as representative as possible. Additionally, while there is potential for selection bias with online surveys, this risk was mitigated by not specifying the topic of the survey in adolescent/caregiver email invitations and carefully designing screening questions to ensure that adolescents and caregivers did not know the purpose of the study until they had met the qualification criteria.

## 5. Conclusions

This analysis of the Spanish cohort of the cross-sectional ACTION Teens survey study highlights existing barriers to effective obesity management of ALwO in Spain, as well as Spanish culture-specific factors. It revealed misperceptions of weight status in a high proportion of ALwO and caregivers of ALwO, and suggested that ALwO are more likely to worry about their weight than their caregivers. Both ALwO and caregivers reported not being able to control hunger as an important barrier to weight loss, while HCPs were more likely to report a poor diet or a lack of physical activity. In addition, communication difficulties and misalignments exist between HCPs, ALwO, and caregivers. HCPs assume that ALwO will not feel comfortable starting conversations about their weight, and a minority of HCPs perceive themselves as being responsible for initiating weight-related discussions. Meanwhile, there is a willingness among both ALwO and caregivers to talk with an HCP about the ALwO’s weight; they feel comfortable discussing it and feel positive after having the conversation. Improved communication between ALwO, caregivers, and HCPs is required, alongside a better understanding of the existing barriers to weight loss. 

## Figures and Tables

**Table 1:** Demographics and clinical characteristics of the ACTION Teens participants from Spain.

Demographic/Characteristic	Spain ALwO	Spain Caregivers	Spain HCPs
Full-country sample, *n*	648	644	251
Matched pair (ALwO and caregiver), *n* (%)	55 (8)	55 (9)	N/A
Unmatched, *n* (%)	593 (92)	589 (91)	N/A
Age in years, mean (SD)	15.0 (1.8)	40.7 (8.9)	43.2 (11.2)
Female, *n* (%) ^a^	231 (36)	289 (45)	116 (46)
Male, *n* (%) ^a^	417 (64)	355 (55)	135 (54)
BMI classification of ALwO ^b^			
Obesity Class I	83% (*n* = 538)	80% (*n* = 517)	62% (SD: 19.9)
Obesity Class II	8% (*n* = 51)	13% (*n* = 85)	26% (SD: 12.3)
Obesity Class III	9% (*n* = 59)	7% (*n* = 42)	13% (SD: 11.1)
BMI classification of caregivers and HCPs, *n* (%) ^c^			
Underweight (<18.5 kg/m^2^)	N/A	7 (1)	5 (2)
Healthy weight (18.5–24.9 kg/m^2^)	N/A	257 (40)	147 (67)
Overweight (25.0–29.9 kg/m^2^)	N/A	251 (39)	53 (24)
Obesity Class I–III (≥30 kg/m^2^)	N/A	129 (20)	14 (6)

^a^ Percentage of male and female participants is based on ALwO/caregiver Q5 (were you born a male or female?) and HCP Q905 (are you male, female, or other?). ^b^ BMI classification for surveyed ALwO, the ALwO of surveyed caregivers, and the ALwO treated by surveyed HCPs. Obesity Class I = BMI ≥95th percentile for age and sex; Obesity Class II = BMI ≥120% of 95th percentile for age and sex; Obesity Class III = BMI ≥140% of 95th percentile for age and sex. ALwO and caregiver data consist of the percentage (number) of ALwO in each BMI category; HCP data consist of the mean percentage (SD) of their ALwO patients in each BMI category. ^c^ BMI classification of surveyed caregivers and HCPs (*n* = 219 for HCPs). ALwO, adolescents living with obesity; BMI, body mass index; HCP, healthcare professional; N/A, not applicable; SD, standard deviation. Table adapted from [14].

## Data Availability

Data will be shared with bona fide researchers submitting a research proposal approved by the independent review board. Individual participant data will be shared in data sets in a de-identified and anonymized format. Data will be made available after research completion. Information about data access request proposals can be found at novonordisk-trials.com.

## Supplementary Materials

The following supporting information can be downloaded at: https://www.mdpi.com/article/10.3390/nu15133005/s1, Table S1: HCP primary medical specialty and professional experience. Figure S1: Disposition of survey participants from Spain: (A) ALwO and caregivers; (B) HCPs. Figure S2: Sources for information about obesity, healthy lifestyles, weight loss, and weight management: (A) sources used by ALwO and caregivers (≥5% of participants); (B) most important information sources according to ALwO and caregivers (≥5% of participants); (C) sources used by HCPs. Figure S3: HCPs’ attitudes towards weight loss in ALwO. Figure S4: ALwO weight loss barriers according to: (A) ALwO and caregivers; (B) HCPs. Figure S5: Definitions of weight loss success among ALwO, caregivers, and HCPs. Figure S6: Barriers to conversations about weight with HCPs: (A) ALwO- and caregiver-reported barriers; (B) HCP-reported barriers; (C) HCPs’ perception of ALwO barriers. Figure S7: Perceptions of conversations about weight between ALwO and HCPs: (A) who initiated discussions; (B) who should initiate discussions. Figure S8: HCPs’ attitudes towards obesity and ALwO weight management. Figure S9: Weight management methods and recommendations: (A) methods used by ALwO in the past year according to ALwO and caregivers; (B) methods recommended by HCPs; (C) most effective methods for weight management, as perceived by HCPs.
