# Supplementary material for: Attitudes, Behaviors, and Barriers among Adolescents Living with Obesity, Caregivers, and Healthcare Professionals in Spain: ACTION Teens Survey Study
Source: Nutrients. 2023 Jun 30;15(13):3005. doi: 10.3390/nu15133005 (PMC10347057; doi:10.3390/nu15133005)
Supplement: Supplementary file 1 [file nutrients-15-03005-s001.zip › nutrients-2418325-supplementary.pdf]

## Supplementary Material

This document contains supporting information for:

# Attitudes, Behaviors, and Barriers among Adolescents Living with Obesity, Caregivers, and Healthcare Professionals in Spain: ACTION Teens Survey Study

Juan Pedro López Siguero, Marta Ramon-Krauel, Gilberto Pérez López, Maria Victoria Buiza Fernández, Carla Assaf Balut, and Fernando Fernández-Aranda

**Correspondence:** lopez.siguero@gmail.com

## Contents


TABLE S1. HCP primary medical specialty and professional experience .................................... 3

FIGURE S1. Disposition of survey participants from Spain ......................................................... 5

FIGURE S2. Sources for information about obesity, healthy lifestyles, weight loss, and weight management ........................................................................................................................ 7

FIGURE S3. HCPs' attitudes towards weight loss in ALwO ......................................................... 9

FIGURE S4. ALwO weight loss barriers .......................................................................................... 10

FIGURE S5. Definitions of weight loss success among ALwO, caregivers, and HCPs............. 12

FIGURE S6. Barriers to conversations about weight with HCPs ............................................... 14

FIGURE S7. Perceptions of conversations about weight between ALwO and HCPs................. 16

FIGURE S8. HCPs' attitudes towards obesity and ALwO weight management. ........................ 18

FIGURE S9. Weight management methods and recommendations .......................................... 19

## Supplementary tables

**TABLE S1.** HCP primary medical specialty and professional experience.

| | **Spain HCPs (n = 251)** |
|---|---|
| **Primary medical specialty, n (%)** [a] | |
| Primary care practitioner | 105 (42) |
| General practice physician | 27 (11) |
| Family practice physician | 72 (29) |
| Internal medicine (primary care practitioner) | 6 (2) |
| General pediatrician | 76 (30) |
| Other specialty | 70 (28) |
| Pediatric endocrinologist | 48 (19) |
| Nutrition specialist | 2 (1) |
| Pediatric gastroenterologist | N/A |
| Psychiatrist | 18 (7) |
| Obstetrician/gynecologist | N/A |
| Internal medicine (specialist) | 2 (1) |
| Pediatric cardiologist | N/A |
| **Professional experience** | |
| Proportion of professional time, mean (SD) | |
| Patient care/medical management | 83 (12.7) |
| Surgical procedures | 3 (6.0) |
| Office procedures (endoscopies, imaging, etc.) | 6 (7.1) |
| Research or administrative tasks | 8 (6.4) |
| Years in practice, mean (SD) | 14.5 (8.4) |
| 2 to <5 years, n (%) | 21 (8) |
| 5 to <15 years, n (%) | 115 (46) |
| 15 to <25 years, n (%) | 70 (28) |
| 25 to <35 years, n (%) | 40 (16) |
| 35+ years, n (%) | 5 (2) |
| Provides obesity/weight management as a primary treatment objective, n (%) | 218 (87) |

| | |
|---|---|
| **Experience with ALwO** | |
| Number of adolescent patients with obesity seen per typical month, mean (SD) | 34.1 (27.4) |
| Proportion of adolescent patients with obesity who are being treated by the HCP for obesity, mean (SD) | 65 (29.9) |
| Years providing obesity care/management to adolescents, mean (SD) | 11.1 (7.9) |

[a] Primary medical specialties are grouped into three overarching categories ("primary care practitioner", "general pediatrician", and "other specialty") – these were not included as prespecified response options in HCP S15, although the "general pediatrician" category was based on "pediatrician" responses. Respondents who selected "internal medicine physician" in HCP S15 were asked to quantify the percentage of time spent in a primary care or family practice setting in HCP S16; responses >50% were coded as "internal medicine (primary care practitioner)" and responses ≤50% were coded as "internal medicine (specialist)".

ALwO, adolescents living with obesity; HCP, healthcare professional; N/A, not appliable; SD, standard deviation.

Table adapted from Halford, J.C.G.; Bereket, A.; Bin-Abbas, B.; Chen, W.; Fernández-Aranda, F.; Garibay Nieto, N.; López Siguero, J.P.; Maffeis, C.; Mooney, V.; Osorto, C.K.; et al. Misalignment among adolescents living with obesity, caregivers, and healthcare professionals: ACTION Teens global survey study. *Pediatr. Obes.* **2022**, *17*, e12957, https://doi.org/10.1111/ijpo.12957.

## Supplementary figures

**FIGURE S1.** Disposition of survey participants from Spain: (A) ALwO and caregivers; (B) HCPs.

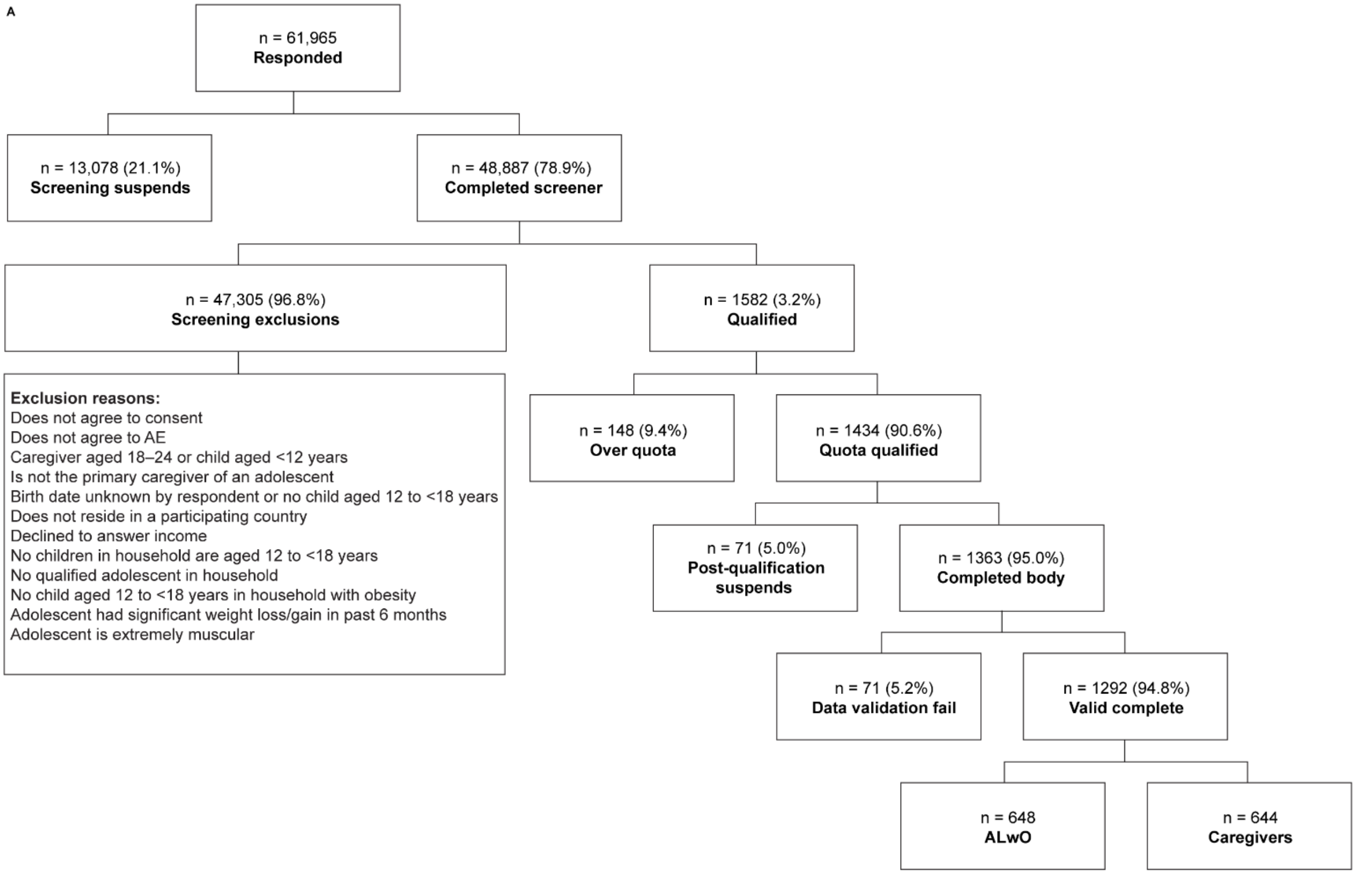

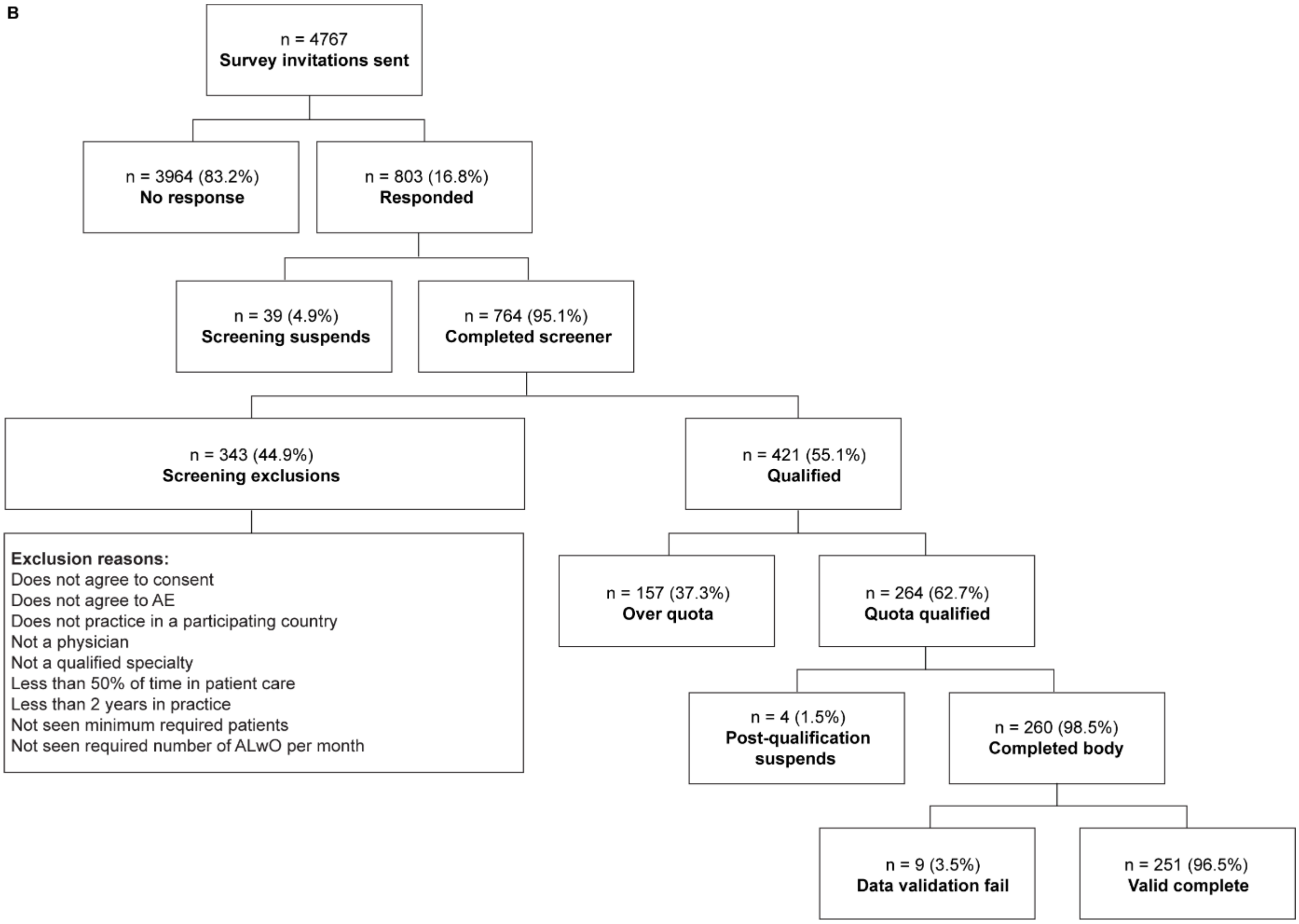


Screening suspends indicates that the respondent failed to complete the qualification section of the online survey, but was not marked as disqualified (i.e., screening drop-out). Over quota indicates that the respondent could not continue because the required number of completed interviews that matched the respondent's qualification criteria had already been collected. Post-qualification suspends indicates that the respondent failed to fully complete the main body of the survey (i.e., survey drop-out). Data validation fail indicates that the respondent's data were removed from the final dataset for failing data validation checks (e.g., providing incorrect responses to data validity questions, having an extremely short completion time, and for "straight-lining" rating scale questions).

AE, adverse event; ALwO, adolescents living with obesity; HCP, healthcare professional.

Figure adapted from Halford, J.C.G.; Bereket, A.; Bin-Abbas, B.; Chen, W.; Fernández-Aranda, F.; Garibay Nieto, N.; López Siguero, J.P.; Maffeis, C.; Mooney, V.; Osorto, C.K.; et al. Misalignment among adolescents living with obesity, caregivers, and healthcare professionals: ACTION Teens global survey study. *Pediatr. Obes.* **2022**, *17*, e12957, https://doi.org/10.1111/ijpo.1295

**FIGURE S2.** Sources for information about obesity, healthy lifestyles, weight loss, and weight management: (A) sources used by ALwO and caregivers (≥5% of participants); (B) most important information sources according to ALwO and caregivers (≥5% of participants); (C) sources used by HCPs.

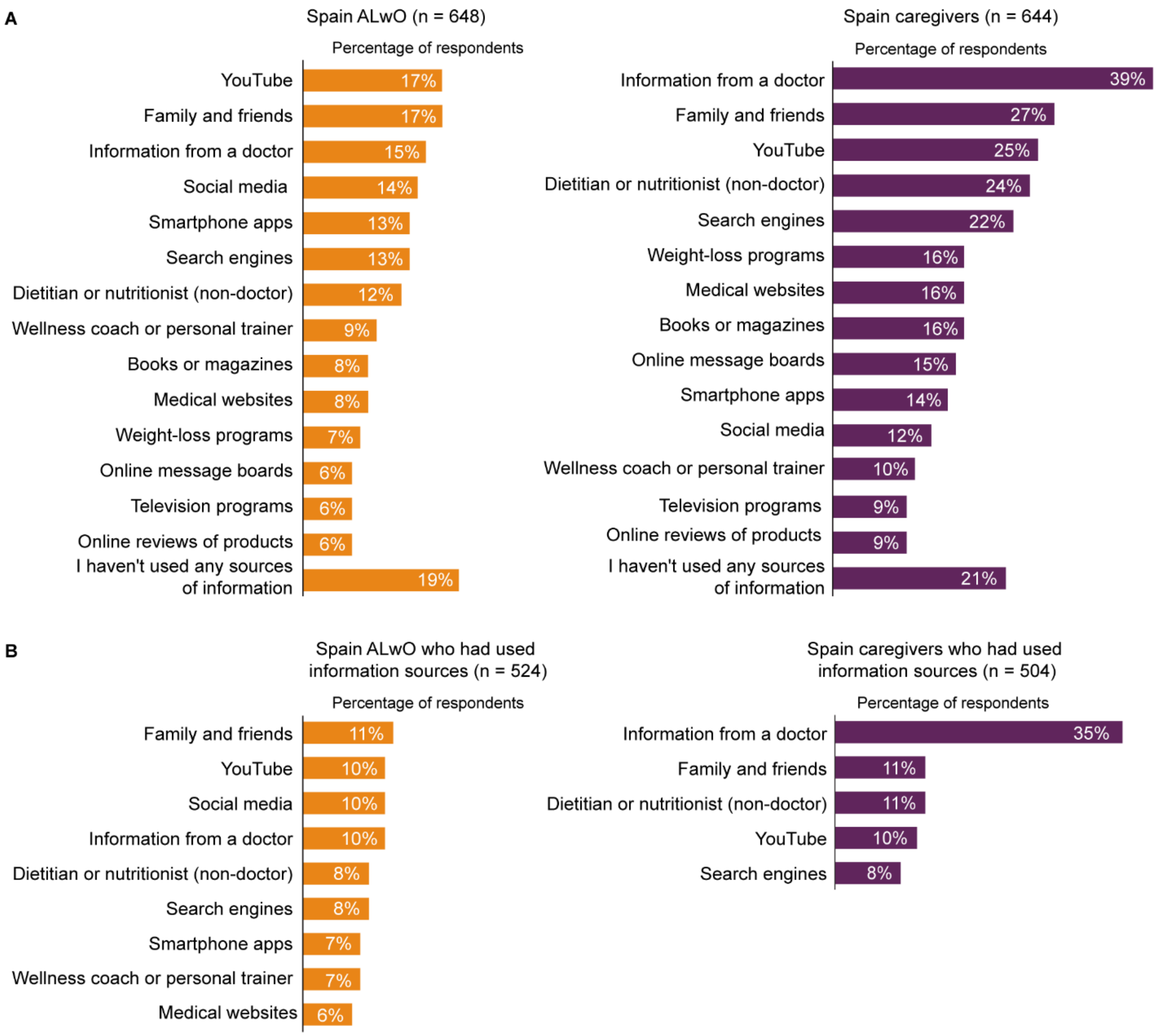

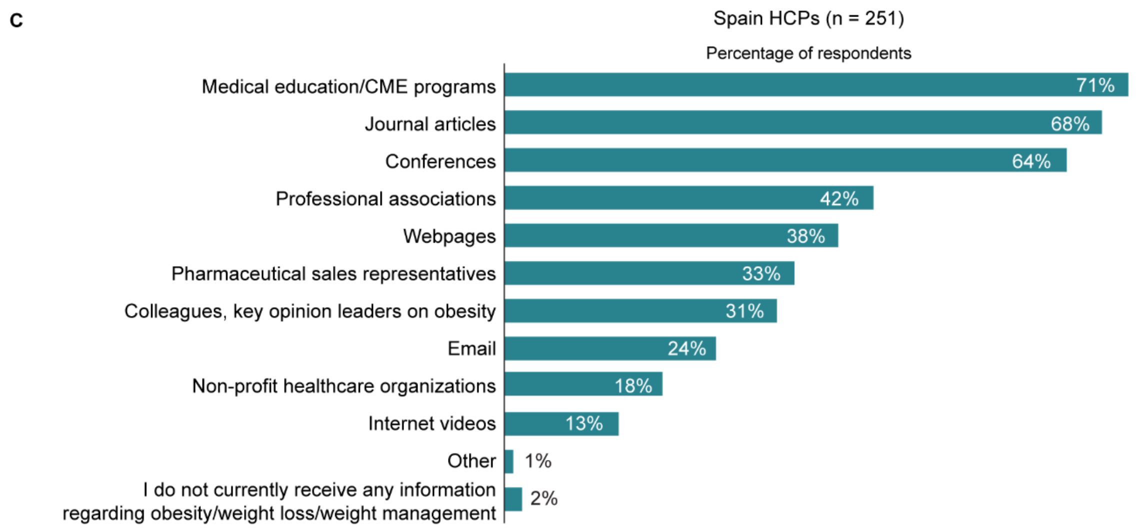


Panel A presents the sources that ALwO and caregivers reported having used from the prespecified list of information sources in AlwO/caregiver Q600, among all recruited AlwO and caregivers. Panel B presents sources that AlwO and caregivers indicated were their single most important source of information, from the prespecified list of sources in AlwO/caregiver Q605, among the AlwO and caregivers who reported using information sources (per ALwO/caregiver Q600). Panel C presents sources that HCPs reported they use to receive information, from the prespecified list of sources in HCP Q505, among all recruited HCPs.

AlwO, adolescents living with obesity; CME, continuing medical education; HCP, healthcare professional.

Figure adapted from Halford, J.C.G.; Bereket, A.; Bin-Abbas, B.; Chen, W.; Fernández-Aranda, F.; Garibay Nieto, N.; López Siguero, J.P.; Maffeis, C.; Mooney, V.; Osorto, C.K.; et al. Misalignment among adolescents living with obesity, caregivers, and healthcare professionals: ACTION Teens global survey study. *Pediatr. Obes.* **2022**, *17*, e12957, https://doi.org/10.1111/ijpo.12957.

**FIGURE S3.** HCPs' attitudes towards weight loss in ALwO.

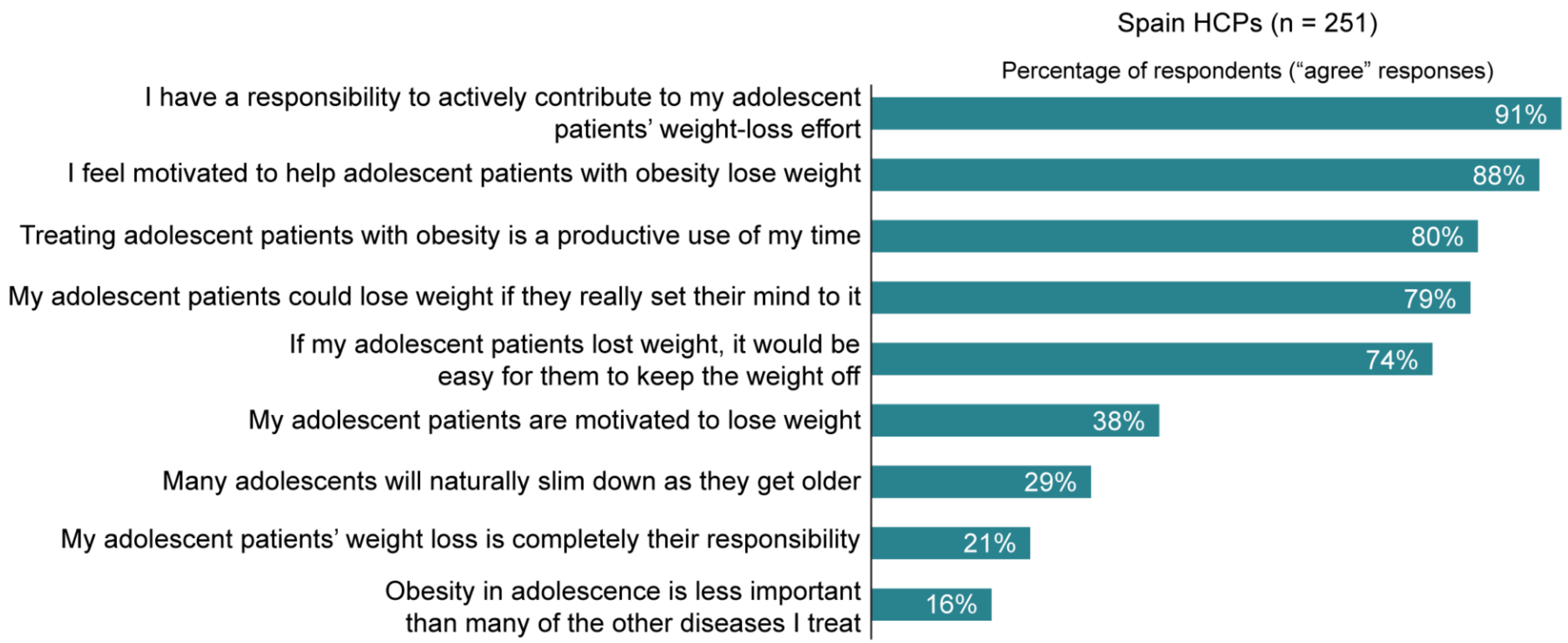


Percentages are proportions of participants who reported that they "somewhat" or "strongly" agree with each statement in HCP Q420, among all recruited HCPs.

ALwO, adolescents living with obesity; HCP, healthcare professional.

Figure adapted from Halford, J.C.G.; Bereket, A.; Bin-Abbas, B.; Chen, W.; Fernández-Aranda, F.; Garibay Nieto, N.; López Siguero, J.P.; Maffeis, C.; Mooney, V.; Osorto, C.K.; et al. Misalignment among adolescents living with obesity, caregivers, and healthcare professionals: ACTION Teens global survey study. *Pediatr. Obes.* **2022**, *17*, e12957, https://doi.org/10.1111/ijpo.12957.

**FIGURE S4.** ALwO weight loss barriers according to: (A) ALwO and caregivers; (B) HCPs.

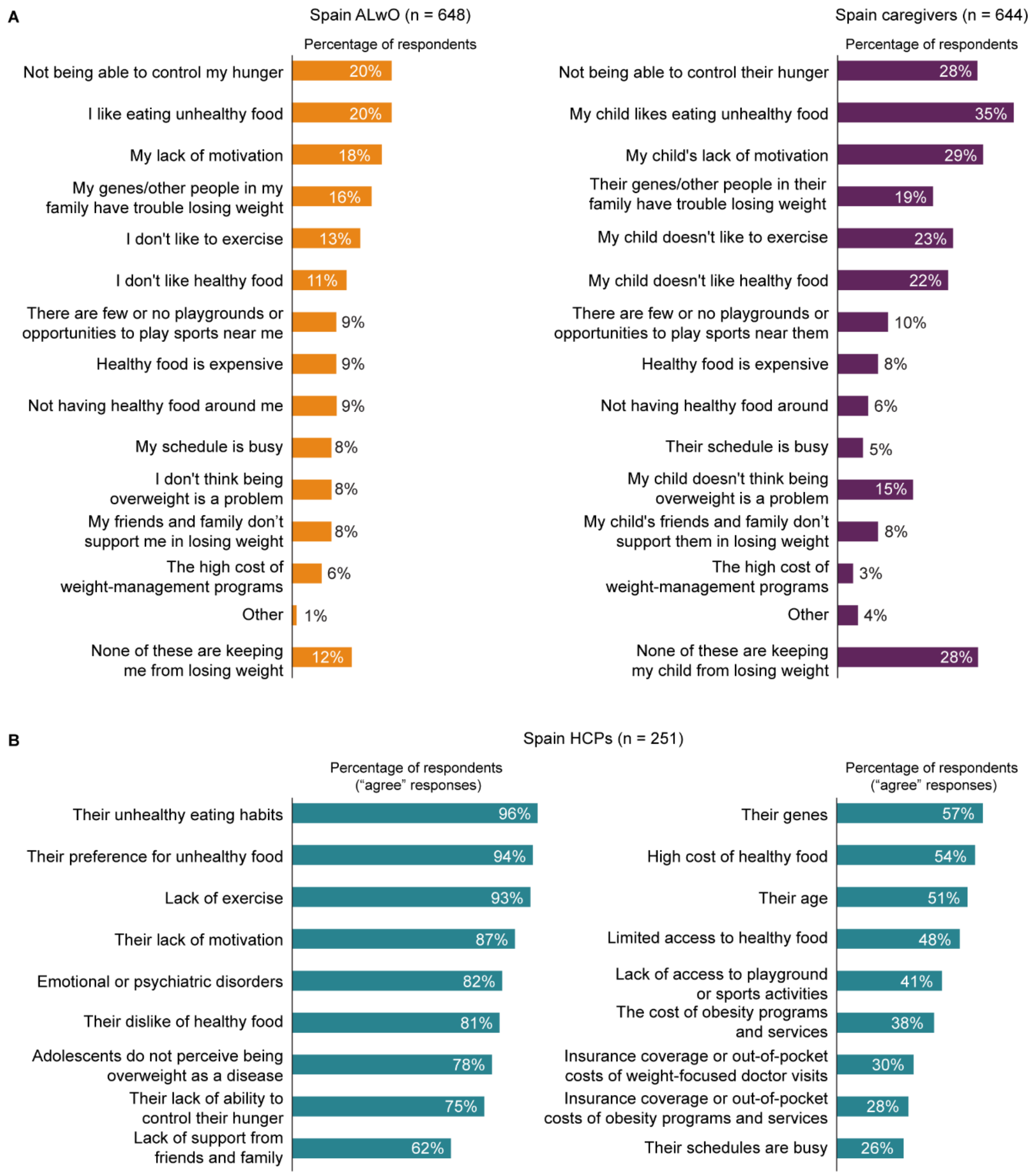

In panel A, percentages are proportions of participants who indicated each statement is a barrier to losing weight for themself (for ALwO) or their child (for caregivers) in ALwO/caregiver Q210, among all recruited ALwO and caregivers. In panel B, percentages are proportions of participants who indicated that they “somewhat agree” or “strongly agree” that each statement is a barrier to their ALwO patients losing weight in HCP Q206, among all recruited HCPs.

ALwO, adolescents living with obesity; HCP, healthcare professional.

Figure adapted from Halford, J.C.G.; Bereket, A.; Bin-Abbas, B.; Chen, W.; Fernández-Aranda, F.; Garibay Nieto, N.; López Siguero, J.P.; Maffeis, C.; Mooney, V.; Osorto, C.K.; et al. Misalignment among adolescents living with obesity, caregivers, and healthcare professionals: ACTION Teens global survey study. *Pediatr. Obes.* **2022**, *17*, e12957, https://doi.org/10.1111/ijpo.12957.

**FIGURE S5.** Definitions of weight loss success among ALwO, caregivers, and HCPs.

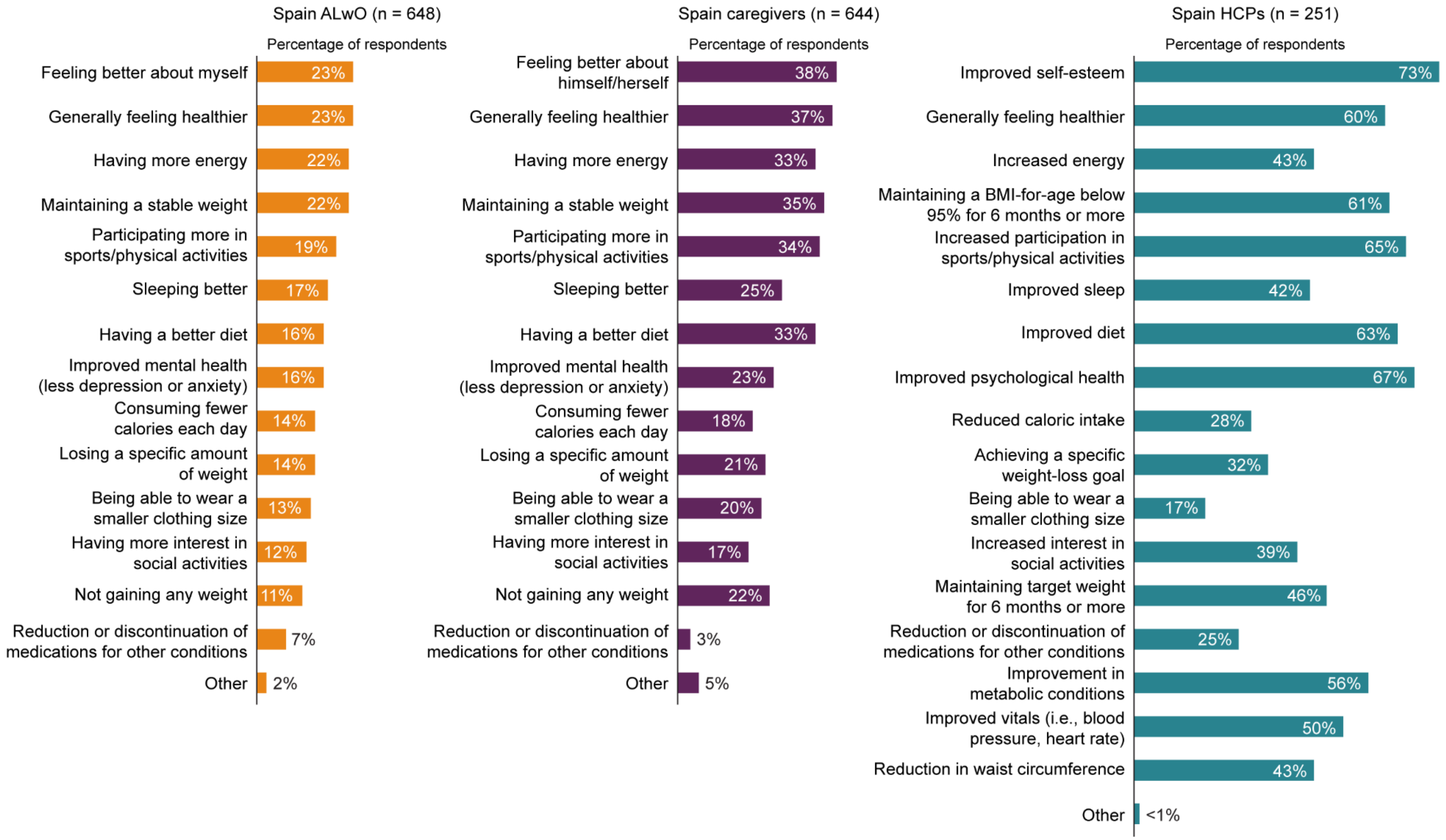

Percentages are proportions of participants who indicated each statement is a definition for “success” when losing weight for themself (for ALwO), their child (for caregivers), or their ALwO patients (for HCPs) in ALwO/caregiver Q220 or HCP Q220, among all recruited ALwO, caregivers, and HCPs. HCP statements are not exact matches to the ALwO and caregiver statements.

ALwO, adolescents living with obesity; BMI, body mass index; HCP, healthcare professional.

Figure adapted from Halford, J.C.G.; Bereket, A.; Bin-Abbas, B.; Chen, W.; Fernández-Aranda, F.; Garibay Nieto, N.; López Siguero, J.P.; Maffeis, C.; Mooney, V.; Osorto, C.K.; et al. Misalignment among adolescents living with obesity, caregivers, and healthcare professionals: ACTION Teens global survey study. *Pediatr. Obes.* **2022**, *17*, e12957, https://doi.org/10.1111/ijpo.12957.

**FIGURE S6.** Barriers to conversations about weight with HCPs: (A) ALwO- and caregiver-reported barriers; (B) HCP-reported barriers; (C) HCPs' perception of ALwO barriers.

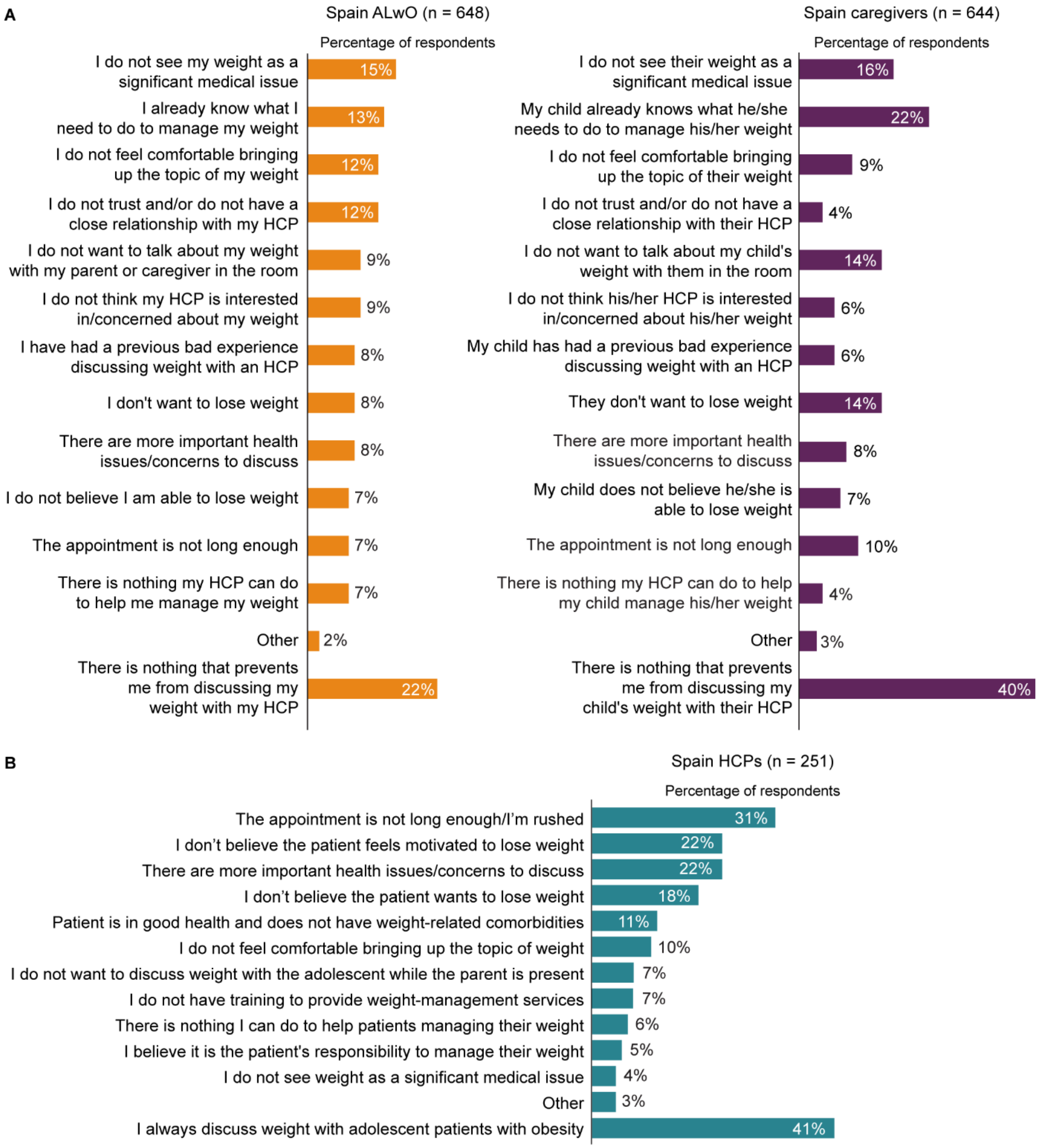

C

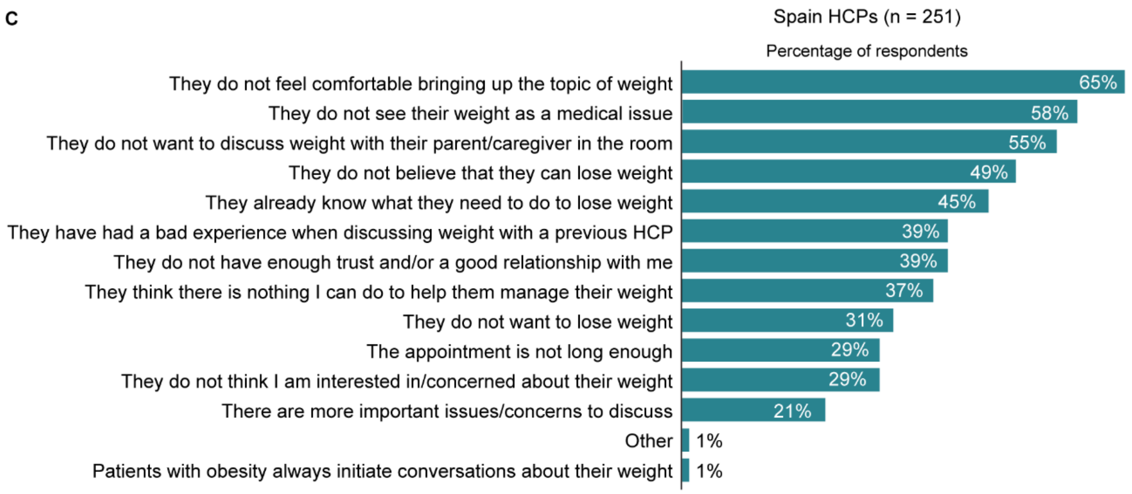


In panel A, percentages are proportions of participants who indicated each statement is a reason for not discussing their (for ALwO), or their child's (for caregivers) weight with the ALwO's HCP in ALwO/caregiver Q425, among all recruited ALwO and caregivers. In panel B, percentages are proportions of participants who indicated each statement is a reason they (i.e., the HCP) might not discuss weight with an ALwO patient in HCP Q413, among all recruited HCPs. In panel C, percentages are proportions of participants who indicated each statement is a reason their ALwO patients might not initiate conversations about their weight in HCP Q412, among all recruited HCPs.

ALwO, adolescents living with obesity; HCP, healthcare professional.

Figure adapted from Halford, J.C.G.; Bereket, A.; Bin-Abbas, B.; Chen, W.; Fernández-Aranda, F.; Garibay Nieto, N.; López Siguero, J.P.; Maffeis, C.; Mooney, V.; Osorto, C.K.; et al. Misalignment among adolescents living with obesity, caregivers, and healthcare professionals: ACTION Teens global survey study. *Pediatr. Obes.* **2022**, *17*, e12957, https://doi.org/10.1111/ijpo.12957.

**FIGURE S7.** Perceptions of conversations about weight between ALwO and HCPs: (A) who initiated discussions; (B) who should initiate discussions.

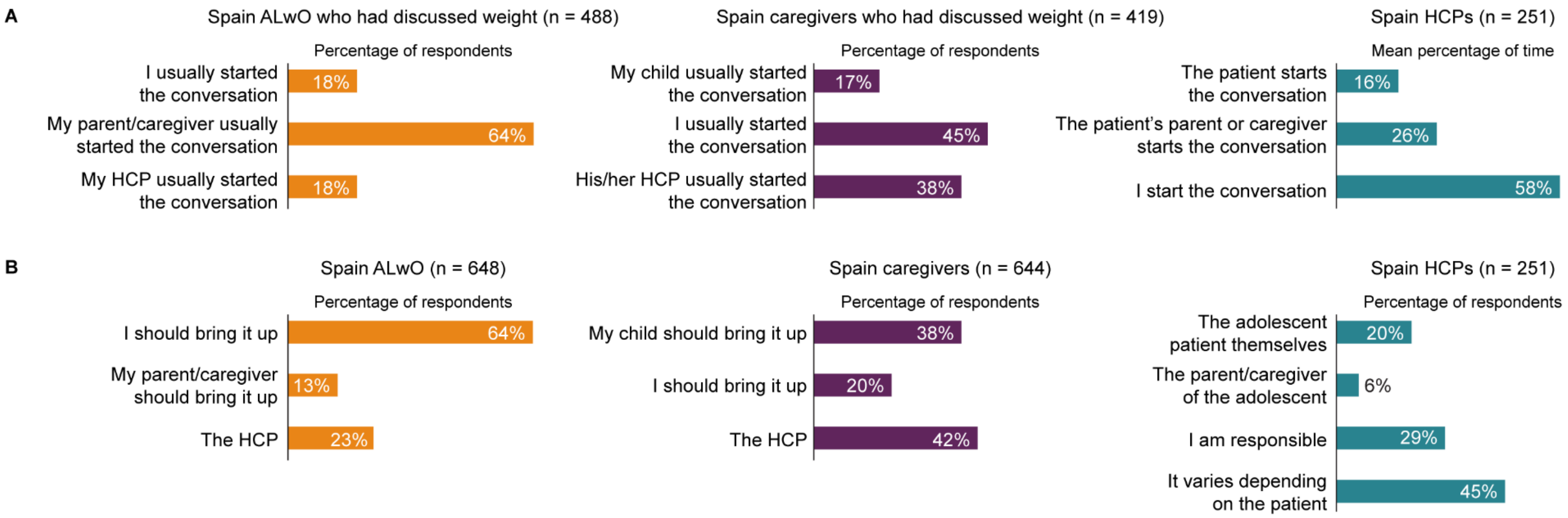


In panel A, ALwO and caregiver data are proportions of participants who selected each of the prespecified response options in ALwO/caregiver Q403 when asked who usually first brought up the topic of weight during HCP appointments, among the subset of ALwO or caregivers who had discussed their (for ALwO) or their child's (for caregivers) weight with an HCP in the past year (per ALwO/caregiver Q201). Only one response option could be selected. HCP data are the mean percentage of time that each group initiates the conversation according to HCPs (per HCP Q403), among all recruited HCPs. In panel B, percentages are proportions of participants who selected each of the prespecified response options in ALwO/caregiver Q403a or HCP Q404 when asked who should bring up the topic of weight, among all recruited ALwO, caregivers, or HCPs. Only one response option could be selected.

ALwO, adolescents living with obesity; HCP, healthcare professional.

Figure adapted from Halford, J.C.G.; Bereket, A.; Bin-Abbas, B.; Chen, W.; Fernández-Aranda, F.; Garibay Nieto, N.; López Siguero, J.P.; Maffeis, C.; Mooney, V.; Osorto, C.K.; et al. Misalignment among adolescents living with obesity, caregivers, and healthcare professionals: ACTION Teens global survey study. *Pediatr. Obes.* **2022**, *17*, e12957, https://doi.org/10.1111/ijpo.12957.

**FIGURE S8.** HCPs’ attitudes towards obesity and ALwO weight management.

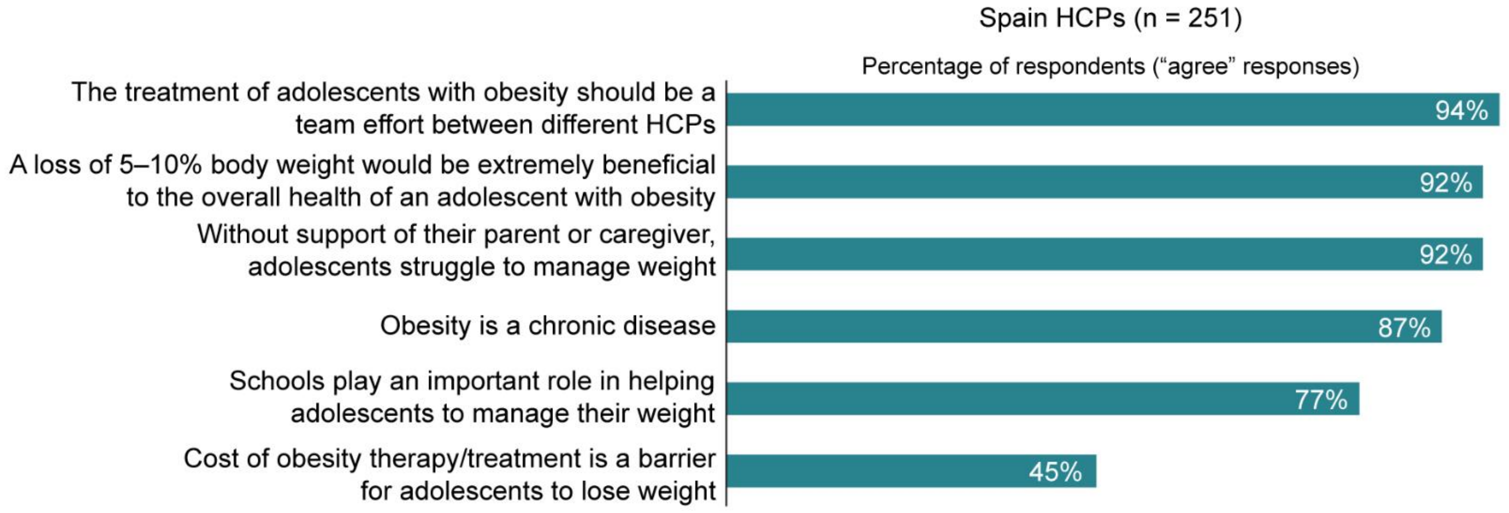


Percentages are proportions of HCPs who indicated that they “somewhat agree” or “strongly agree” with each statement in HCP Q320, among all recruited HCPs.

ALwO, adolescents living with obesity; HCP, healthcare professional.

Figure adapted from Halford, J.C.G.; Bereket, A.; Bin-Abbas, B.; Chen, W.; Fernández-Aranda, F.; Garibay Nieto, N.; López Siguero, J.P.; Maffeis, C.; Mooney, V.; Osorto, C.K.; et al. Misalignment among adolescents living with obesity, caregivers, and healthcare professionals: ACTION Teens global survey study. *Pediatr. Obes.* **2022**, *17*, e12957, https://doi.org/10.1111/ijpo.12957.

**FIGURE S9.** Weight-management methods and recommendations: (A) methods used by ALwO in the past year according to ALwO and caregivers; (B) methods recommended by HCPs; (C) most effective methods for weight management, as perceived by HCPs.

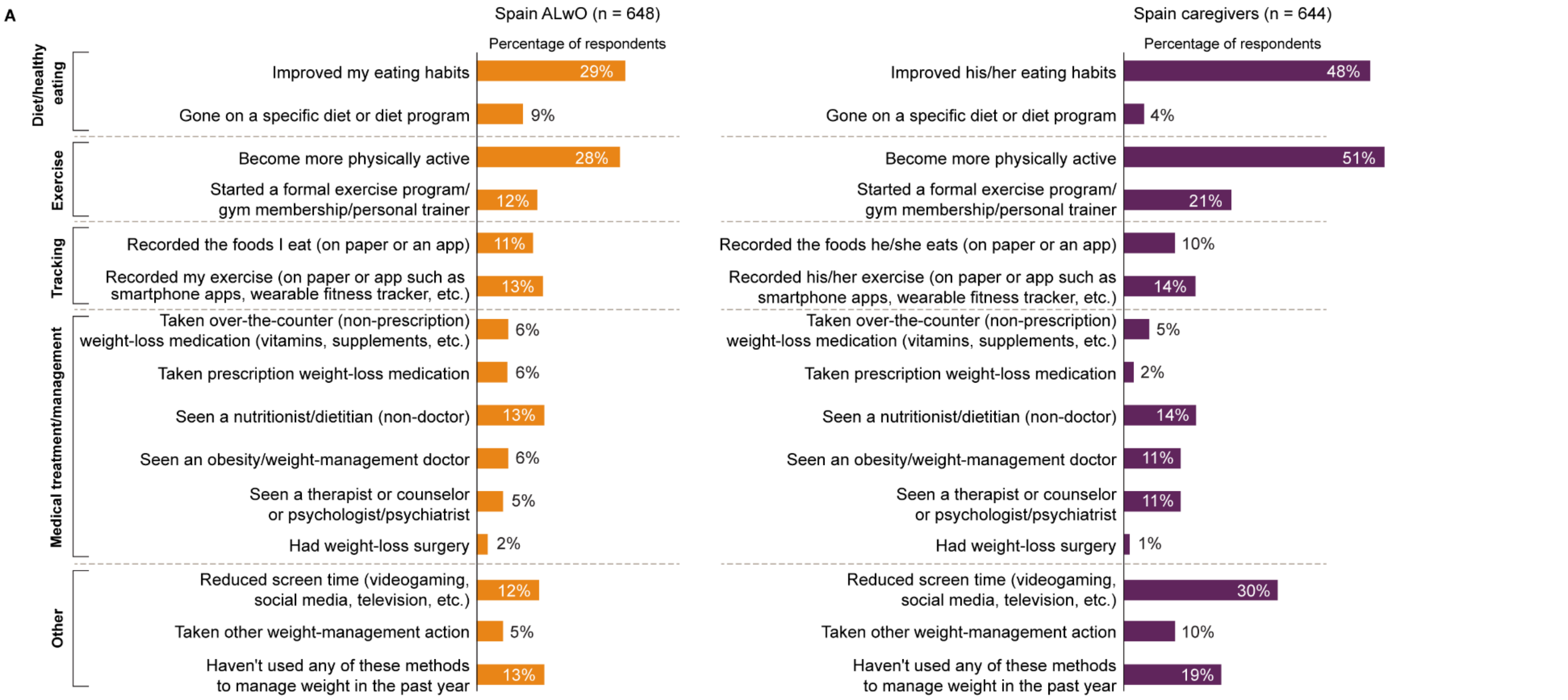

B

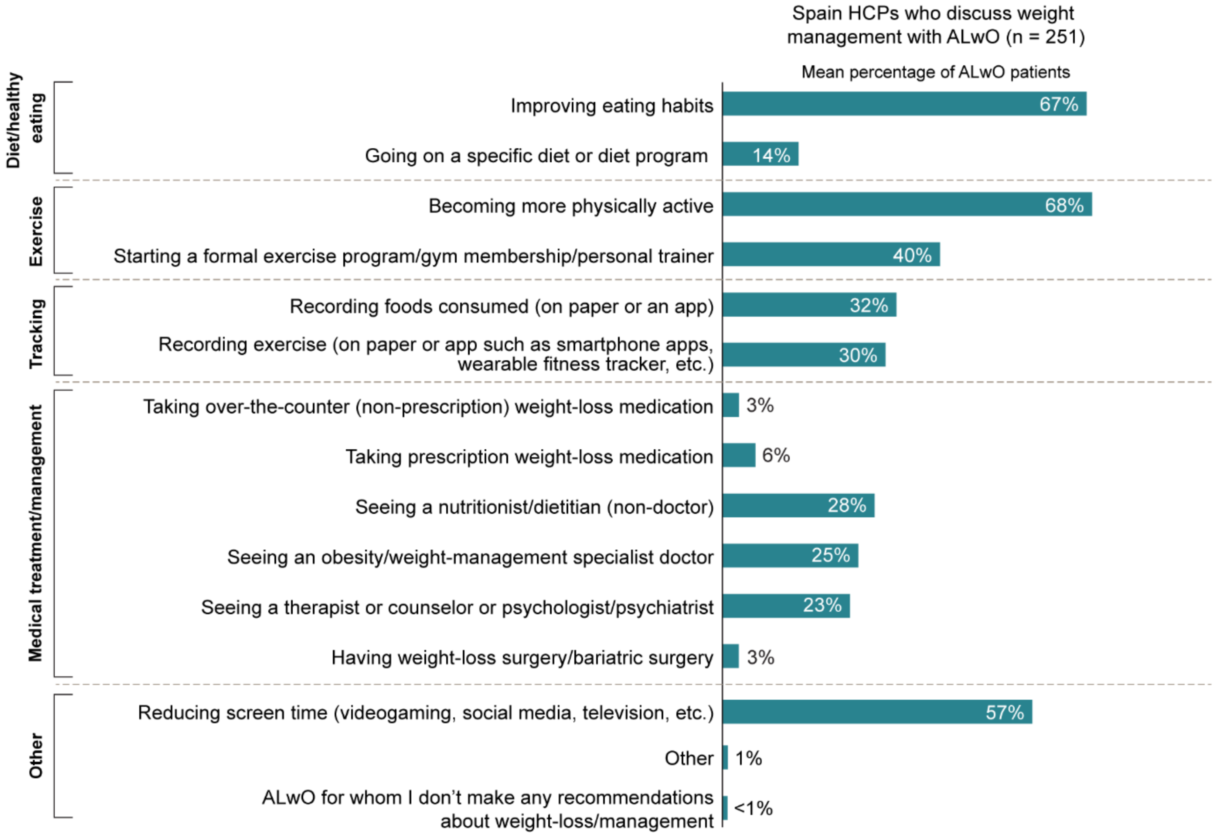
Spain HCPs who discuss weight management with ALwO (n = 251)
Mean percentage of ALwO patients
Diet/healthy eating
Improving eating habits
67%
Going on a specific diet or diet program
14%
Exercise
Becoming more physically active
68%
Starting a formal exercise program/gym membership/personal trainer
40%
Tracking
Recording foods consumed (on paper or an app)
32%
Recording exercise (on paper or app such as smartphone apps, wearable fitness tracker, etc.)
30%
Medical treatment/management
Taking over-the-counter (non-prescription) weight-loss medication
3%
Taking prescription weight-loss medication
6%
Seeing a nutritionist/dietitian (non-doctor)
28%
Seeing an obesity/weight-management specialist doctor
25%
Seeing a therapist or counselor or psychologist/psychiatrist
23%
Having weight-loss surgery/bariatric surgery
3%
Other
Reducing screen time (videogaming, social media, television, etc.)
57%
Other
1%
ALwO for whom I don’t make any recommendations about weight-loss/management
<1%

C

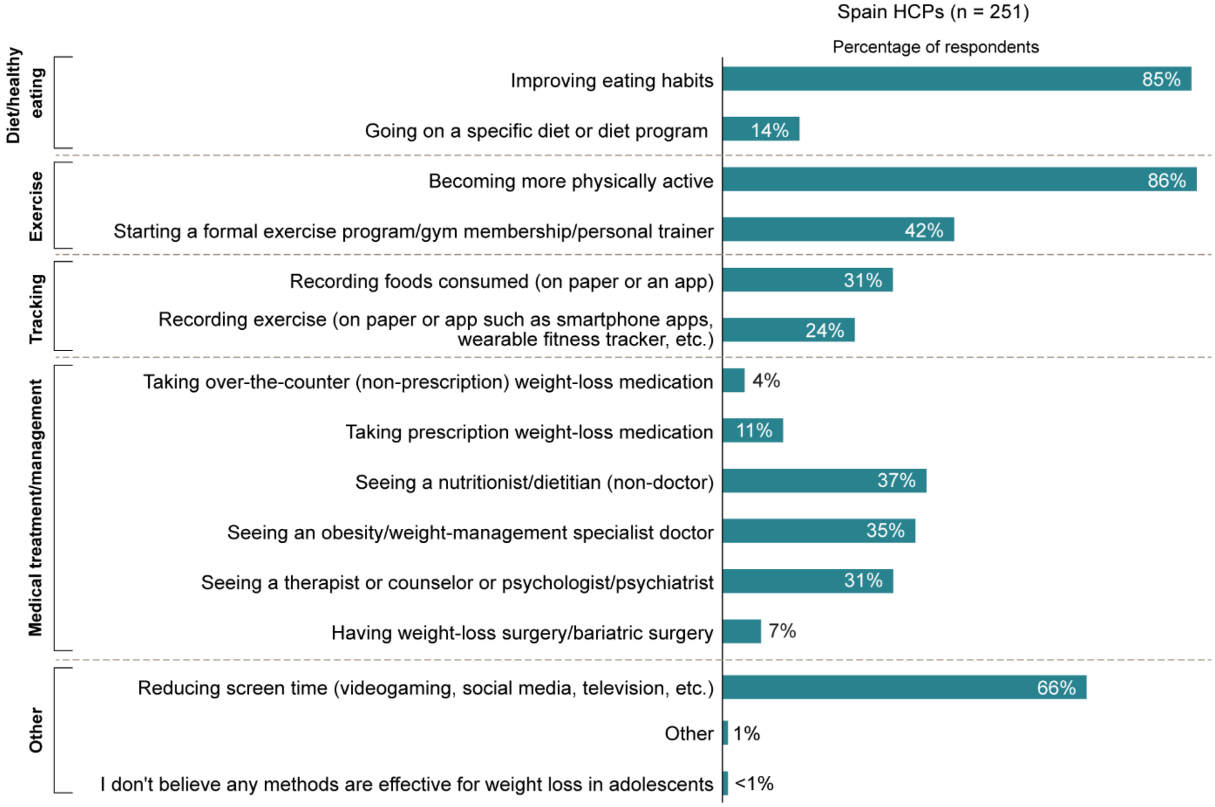
Spain HCPs (n = 251)
Percentage of respondents
Diet/healthy eating
Improving eating habits
85%
Going on a specific diet or diet program
14%
Exercise
Becoming more physically active
86%
Starting a formal exercise program/gym membership/personal trainer
42%
Tracking
Recording foods consumed (on paper or an app)
31%
Recording exercise (on paper or app such as smartphone apps, wearable fitness tracker, etc.)
24%
Medical treatment/management
Taking over-the-counter (non-prescription) weight-loss medication
4%
Taking prescription weight-loss medication
11%
Seeing a nutritionist/dietitian (non-doctor)
37%
Seeing an obesity/weight-management specialist doctor
35%
Seeing a therapist or counselor or psychologist/psychiatrist
31%
Having weight-loss surgery/bariatric surgery
7%
Other
Reducing screen time (videogaming, social media, television, etc.)
66%
Other
1%
I don't believe any methods are effective for weight loss in adolescents
<1%

In panel A, percentages are proportions of participants who selected each of the prespecified response options in ALwO/caregiver Q205 when asked which methods they (for ALwO) or their child (for caregiver) had used in the past year, among all recruited ALwO and caregivers. In panel B, percentages are the mean percentage of ALwO patients that HCPs recommend/prescribe each of the prespecified options in HCP Q212 to, among the subset of HCPs who had discussed weight management with their ALwO patients (per HCP Q210). In panel C, percentages are proportions of HCPs who selected each of the prespecified response options in HCP Q213 when asked which methods of weight management are most effective in ALwO, among all recruited HCPs.

ALwO, adolescents living with obesity; HCP, healthcare professional.

Figure adapted from Halford, J.C.G.; Bereket, A.; Bin-Abbas, B.; Chen, W.; Fernández-Aranda, F.; Garibay Nieto, N.; López Siguero, J.P.; Maffeis, C.; Mooney, V.; Osorto, C.K.; et al. Misalignment among adolescents living with obesity, caregivers, and healthcare professionals: ACTION Teens global survey study. *Pediatr. Obes.* **2022**, *17*, e12957, https://doi.org/10.1111/ijpo.12957.
